# Asterias Rubens

**Published:** 1888-01

**Authors:** George H. Clark

**Affiliations:** Germantown, Philadelphia


### ASTERIAS RUBENS.

GEORGE H. CLARK, M. D., GERMANTOWN, PHILADELPHIA.

A remedy that gives in its pathogenesis symptoms similar to apoplexy, cancer, epilepsy, hysteria, and various uterine affections is certainly entitled to marked notice. As Asterias rubens does possess symptoms similar to the above affections the time devoted to its study should be profitable.

As to symptoms pertaining to apoplexy we find: Became very impatient, which is a precursor of that affection. Sudden attacks of vertigo, like shocks in the head. Sanguineous congestion to the brain.

A prominent symptom is: Wakes at night with sensation as if the brain were shaken by electric shocks; head seems empty; almost deprived of consciousness; thinks he is attacked by apoplexy; lasts several minutes; when he recovers consciousness pulse hard. Again, this symptom was somewhat modified; there was the awakening at night in distress as from electric shocks in the brain; head felt as if bursting; apprehended apoplexy; when he came to himself pulse was hard, very quick, and the right parotid throbbed violently.

There are cerebral congestions, accompanied by obstinate constipation. The outer head is burning.

Another prominent symptom of apoplexy: Gradually lost his sight; pupils were closed by the excessively contracted irides. During the night great agitation and little sleep. The face is red, pulse hard, compressed, and frequent, and where can a more perfect picture of cancer be found, particularly the glandular form, scirrhus? Earthy appearance of face. Obstinate constipation; twelve to fifteen days elapsed without an evacuation, which consisted, when it occurred, of very hard, round substances about the size of an olive. A symptom of cancer of the rectum.

Lancinating pain in the mammary tumor. Lancinating or acute smarting pains entirely deprived her of rest, especially at night. Drawing pain in the breasts. Left breast feels drawn in. Around the nipple, which was sunk into a cavity, skin smooth and adherent; upon one point a violet spot, having fungus hematodes. Ulceration and swelling in left breast; sharp, stitching pain going through to back.

Breasts swollen, distended as before the menses, induration of left mamma, size of head of infant; insensible, hard, and angular. Right breast beginning to manifest symptoms of scirrhus.

A scirrhus tumor forms in right mamma, adhering by its entire base to the thoracic walls. A livid red spot appeared upon one point of the tumor, broke, and gave exit to a discharge; gradually invaded the whole breast, eight inches in circumference, discharging very fetid ichor; edges pale, elevated, mamillary, hard, everted; bottom covered with reddish granulations.

Sternal integument swollen and painful. Axillary glands swollen, hard, and knotted. Nocturnal lancinating pain in the tumor. Ulcers with sensitive edges, fetid discharge.

This remedy is rich in symptoms of epilepsy. There are: Loss of consciousness. Does not lose consciousness but has hallucinations, as if away from home in the midst of strangers; hears voices to which he replies. Easily excited by any emotion, especially by contradiction. A violent pressure upon the anterior lobes of the brain, extending even beneath the eyes; one day so severe that while seated at the table she fell forward, and remained unconscious for some minutes.

Pallor of the face. Her face was pale, and her jaws set while unconscious. At the commencement of the last meal of the day falls forward unconscious. Great debility, with distress in the epigastrium. With the attacks convulsive motions of the limbs. Twitching over the whole body four or five days before the attacks. Weak and pale. Sudden falling; livid face, convulsive motion of the jaws, froth at the mouth, shocks in the limbs, loss of consciousness. After the attacks great debility, with a sense of distress in the epigastrium.

Convulsive attacks, after pressing pain in forepart of the head and over the eyes, with falling forward, loss of consciousness, trismus, pale face, convulsive motion of the limbs. After attacks, prostration and anxiety in upper part of abdomen.

For hysterical symptoms we have: It seems as if some misfortune were impending, as if bad news were about to arrive; tears afford relief, and the various uterine symptoms, which account for the hysterical condition.

There is excitement of the venereal appetite, in the morning in bed, not removed by coitus; annoying, making her ill-humored and disposed to weep.

Sensation of pressure on the lower abdominal organs impeding locomotion.

Venereal desires, erotic thoughts, nervous agitation and distress produced by importunate venereal craving resembling an irresistible power, giving rise to ideas of violence, despair, etc. General feeling of distress in the womb, as though something were passing out.

Jerking in the uterus. Severe general pain over the womb, as if something protruded behind it. Sensation in the womb, as if something was pushing.

Menses are delayed, and colic and other sufferings cease with the flow, which is more abundant than usual, and there is drawing pain in the back and sacrum.

Closely related to Ast. rub. are Murex and Sepia.

Murex produces a depressing effect upon the sensorium, and there is confusion of ideas and diminished intellectual activity.

The provings of Murex are not yet sufficiently extended to show its full value, but like Ast. rub. and Sepia its effects upon the uterus and its appendages are notable. It produces sexual excitement, which is so evident as to fatigue the reason; being, in this respect, very similar to Ast. rub. Platina has violent sexual excitement. Hyoscyamus, also, but with disturbance and perversion of the intelligence and moral sense constituting nymphomania.

Hyoscyamus is closely allied to Ast. rub. in having apoplectic conditions alternating with epilepsy.

In Murex the menses are delayed, with this peculiarity, that after flowing a few days they cease, and after twelve hours re-appear. Sepia, also, has this symptom.

Kreosote has a similar condition, together with irritation of the bladder, and a very acrid discharge from the vagina, causing the pudenda and thighs to swell and become raw, burning and itching.

Murex has severe pains in the mammæ and sharp lancinations, and is in these allied to Ast. rub.

Lilium tigrinum is another remedy worthy of comparison with Ast. rub., while Bell., Calc. carb., Carbo an., Conium, Sil., and Sulph. are remedies which deserve study in the same connection.

